# Comparison of dehydration systems in quality and chemical effects on sunflower seeds

**DOI:** 10.1038/s41598-024-62822-5

**Published:** 2024-05-26

**Authors:** A. A. Ortiz-Hernandez, M. A. Araiza-Esquivel, L. Delgadillo-Ruiz, L. I. Espinosa-Vega, C. A. Olvera-Olvera, A. Lopez-Martinez, H. R. Vega-Carrillo, J. J. Ortega-Sigala

**Affiliations:** 1 Research Department, AORTech, Purísima 227, Lomas de Cristo, 98085 Zacatecas, México; 2 Unidad Académica de Fisica, Benemérita Universidad Autónoma de Zacatecas (BUAZ), Jardín Juárez 147, Centro, 98000 Zacatecas, Mexico; 3 Unidad Académica de Ingeniería Eléctrica, Benemérita Universidad Autónoma de Zacatecas (BUAZ), Jardín Juárez 147, Centro, 98000 Zacatecas, Mexico; 4 Unidad Académica de Ciencias Biologicas, Benemérita Universidad Autónoma de Zacatecas (BUAZ), Campus II UAZ. Avenida Preparatoria S/N. Col. Hidráulica, 98068 Zacatecas, Mexico; 5 Coordinación para la Innovación y Aplicación de la Ciencia y la Tecnología (CIACYT), Universidad Autónoma de San Luis Potosí (UASLP), Av. Sierra Leona #550, Col. Lomas 2a Sección, 78210 San Luis Potosí, Mexico; 6 Unidad Académica de Estudios Nucleares, Benemérita Universidad Autónoma de Zacatecas (BUAZ), Jardín Juárez 147, Centro, 98000 Zacatecas, Mexico

**Keywords:** Proteins, Nutrition, Electrical and electronic engineering

## Abstract

The present work investigates the quality and the chemical effects of dehydration, using a novel dehydration system based on an electromagnetic induction and low pressures technique, comparing it with the thermo-solar drying system. High oleic sunflower seeds, which are an important oil seed crop, were used due to the fact that they have a special place in the food industry. The seed samples were exposed to electromagnetic induction and low pressures by 0.5 and 1 h, then several chemical characterizations were carried out, in the electrophoresis study, it was found that most proteins in the hull were degraded or denatured, some of them were lost during the time in the thermosolar dryer while in kernel keeps 94.9% of the concentration in control proteins. Otherwise, the electromagnetic induction dryer did not lose the most of proteins in the kernel keeping 99.1% in 0.5 h and 98.4% in 1 h, just degrading its concentration. Germination viability results did not show changes after 0.5 h in the electromagnetic fields, but they decreased in 1 h from 66 to 40% until the thermosolar method fell to 24% in 4 h, both analysis results change proportionally with the treatment time and moisture content and the amount of the oxygen.

## Introduction

From 2016 to 2025, according to OCDE-FAO, the worldwide production of cereals and oilseed will increase by 13%. The 1.5% annual growth will be due to the vegetable oils demand^1^. On the other hand, according to USDA, world production is expected to grow by 10.8% from 2020 to 2029, due to the fact that world consumption will increase by 11.4% during the projection period^2^.

To avoid fungi and bacteria, and to be sold at competitive prices, the sunflower seed moisture must be less than 8%^3^. Dehydration can be performed using natural or artificial methods. The drawback of using natural dehydration methods is that these delay the harvest and crops have a larger risk of being impacted by pest or weather changes^4^.

Artificial dehydration methods include Infrared Drying (IRD), Desiccant Drying (DD), Reactance Window Drying (RWD), Supercritical Carbon Dioxide Drying (SCCO_2_D), Superheated Steam Drying (SSD), Heat Pump Drying (HPD), Radio Frequency Drying (RFD), Controlled sudden decompression to vacuum (Dénte Instantanée controlee) (DIC), Ultrasonic Drying (UD), Electric field technologies, for example, Electric Resistance Drying (ERD) or Electrohydrodynamic Drying (ED)^5^ and Thermo-solar drying (TSD) where solar energy is used to heat a flow of air that is used to eliminate the moisture in the samples. Regardless of the drying method the goal is to keep the quality of the product, therefore it is important to analyze the impact of the drying method on the dried product.

Induction heating is widely used, mainly in metalworking industry applications because it is a fast, precise, and repeatable non-contact method. It has been used to heat metals from 50 to 3000 °C^6–11^. Electromagnetic Induction is used by very short time lapses of 0.5 s to include months of heating processes^11^. Their variety of applications includes cooking metal melting, heat treatments, and welding^8^. However, induction heating has not been very used for dehydration applications^3^.

The dehydration procedure can have a negative impact on the seeds ranging from morphological changes to modifications in their chemical features. These drawbacks should be considered when any dehydration method is selected.

Highlights of the research paper previous Physical changes of sunflower seeds dehydrated by using electromagnetic induction and low pressures^4^, the dryer systems proposed (DEMI-LP) was 2.5 times shorter, Several interesting physical effects in seeds and kernels were monitored in this work: Finding an increment of 5% volumetric expansion coefficient, drying sunflower seeds reduces cut resistance from 2.5 to 1.5 Kgf, the porosity increases from 34.3 to 43.3% and from 45.4 to 50.2% (seeds and kernels), the spores of Aspergillus spp, disappear when are dehydrated by DEMI-LP method, represents significant advantages for the food dryer industry.

This work builds on a previous study aiming to compare two dehydration methods; thus, the objective of this work is to compare the effect of TSD and the Dryer with Electromagnetic Induction and Low Pressures (DEMI-LP) on the morphological changes, germination viability, bromatological features, mineral concentrations, densitometry and protein content of sunflower seeds.

## Materials and methods

### Biological material

The “Hi-Oleic” variety sunflower of the (*Helianthus annuus* species), is widely grown in Mexico taking 125 days to mature. For this work, freshly harvested samples of sunflower seeds were used. The samples are the product of the first generation of the Pioneer® P64H41® precocious hybrid. The mean plant height is 111 cm taking 60 days for flowering and 95 days for physiological maturity. The true density of the whole seeds was determined in grams per cubic decimeter (g/dm^3^) by filling a cubic decimeter container with sunflower seeds and averaging the mass measurements obtained three times. The results were reported as ranging from 45.05 g/dm^3^ for control samples with 14.9% *d.b.* moisture to 36.43 g/dm^3^ for samples dehydrated for 1.5 h in DEM-LP^4^. The sunflower seeds were collected approximately 40 km northwest of the capital of the state of Zacatecas (22.98° latitude, 102.28° longitude) in November 2017, when the plants showed all the visual characteristics of physiological maturity. Ten bulk samples (1 kg each) of seeds were collected and transported to the DryLab-AORTech laboratories. Seeds were manually cleaned from foreign matter, eliminating broken or immature seeds.

Subsequently, in 2018, the dried samples underwent comprehensive analysis at the Biochemical Laboratory of the Academic Unit of Biology at the Autonomous University of Zacatecas, to conduct the majority of the analyses reported in this postdoctoral research project.

### Dehydration methods

#### Thermo-solar dehydration (TSD)

The TSD was used to remove the moisture from the samples. This system has a closed chamber with concave solar concentrators where solar-heated air circulates.

#### Dehydration by electromagnetic induction and low-pressures (DEMI-LP)

The dehydration method employed in this study utilized the DEMI-LP device, which is currently pending patent approval^12^. Essentially, this system consists of a gyratory hermetic cylinder crafted from food-grade stainless steel, referred to as the chamber. This chamber is in thermal contact with a martensitic stainless-steel jacket, which is heated through an electromagnetic field operating at the resonance frequency of ferromagnetic materials. Previous research papers have reported on the physical changes induced by this method. Additionally, further details and a schematic diagram were included to enhance functional comprehension^4^. The electromagnetic field whose frequency is the same as the resonance frequency of ferromagnetic materials.

### High-resolution transmission electron microscope

In order to detect morphological changes in the surface of dehydrated seeds, these were analyzed by obtaining micrographs 500× of the surface of the seeds using High-resolution transmission electron microscopy with a JEOL 2010F field emission microscope operating at 200 kV. These micrographs were obtained at the Kleberg Advanced Microscopy Center at The University of Texas at San Antonio.

### Germination viability

In the aim of evaluating the vigor of dehydrated sunflower seeds, the laboratory germination viability was determined. For this, 25 seeds were dehydrated for 0.5 h with the DEMI-LP, 25 seeds were dehydrated for 1.0 h with the DEMI-LP, and 25 seeds were dehydrated for 4 h with the TSD, these methods were randomly selected. As a control, also 25 non-dehydrated seeds were selected. Each set was allocated in a container with 3% of calcium hypochlorite solution, the containers were shaken for 3 min and the hypochlorite solution was eliminated, then the seeds were washed with sterilized water three times. In a germination tray, a hydrated pigment substrate with sterilized water was placed, and each seed was allocated in each well. The trays were kept inside the germination chamber at a controlled temperature of about 25 °C for 15 days. Once the germination time elapsed, the germinated seeds were considered and counted when they presented radicle emergence ISTA (1999)^13^.

### Bromatological analysis

Moisture (M) is inversely proportional to the dry matter (DM) measured on a dry basis (*d.b.*). The DM was measured in seeds dehydrated by both methods using the method of AOAC (1995). Seeds were grounded in a food processor until two-micro size particles were obtained, these were used to determine the crude fiber (*CF*), the ash (*Ash*), the crude protein content (*Pc*), and the ether extract (*EE*). Also, the total reducing sugars were determined using the Lane and Eynon technique^14^. These analyses were carried out three times. The total organic carbon (TOC) was calculated using the Eq. (1).

1 $$TOC=100-\left(\text{\%}\frac{C}{1.8}\right)$$

Here, *C* is the carbon mass.

### Mineral analysis (macro and micro)

The mineral content of dehydrated seeds was carried out using the digestion method where seeds were dried, grinded, pulverized, and allocated into an acid mixture of HNO_3_ and H_2_O_2_ to destroy the organic material. Then, they were digested using microwaves with a Microwave Milestone MLS-1200. Digested material was analyzed by optical emission spectroscopy with inductively coupled plasma ICP-OES (Perkin Elmer Optimus 7000), where was determined the concentration of calcium (Ca), phosphorus (P), magnesium (Mg), potassium (K), copper (Cu), zinc (Zn), manganese (Mn) and iron (Fe). This analysis was performed twice.

### Extraction and quantification of proteins

In order to extract the protein content 500 μg of the sunflower seed was resuspended in 500 μL of a NET-2 extraction buffer (500 mM Tris–HCl pH 7.5, 140 mM NaCl, 0.05% NP-40), then were vortexed and centrifuged to 6000 rpm for 10 min, and the supernatant was removed and transferred to 1.5 mL conical tubes, as described by Nambara et al.^15^. Protein content was determined with a spectrophotometer using the Micro-Bradford technique^16^ and with modifications introduced by Simonian & Smith (2006). In a microtiter plate (microplate),10 μL of the sample was added and the volume was adjusted to 20 μL using a 150 mM NaCl solution. For each well, 180 μL of the Bradford working solution (Bradford Dye Sigma) was added. The absorbance was read at an optical density of 595 nm. Bovine serum albumin was used as a calibration reference^17^, and each determination was made in triplicate.

#### Protein electrophoresis of sunflower seeds tissues in polyacrylamide gels (PAGE-SDS)

The densitometry and the concentration of proteins in seeds dehydrated by TSD and DEMI-LP were determined.

With the protein extracts obtained, the 12% PAGE-SDS polyacrylamide gels were made (Acrylamide-Bis Acrylamide 30:08, tris–HCl 1.5 M pH 8.8, SDS 10%) by conventional methods, and 60 mg of protein per lane was used. These samples were denatured for 5 min with a denaturing buffer (2.5 mL Tris–HCl 0.5 M pH 6.8,1 mL Bis-mercaptoethanol 5%, 4.5 mL distilled H_2_O, 4 mL 10% SDS, 20 μL or 0.0002 g bromophenol blue 1%, 8 mL Glycerol 10%) consisting in tubes placed over boiling water in a hot dish. The gels were run for 30 min at 80 V and then for 120 min at 100 V. Once the shift was over, the gels were stained with Coomassie blue *G-250* (C_47_ H_48_ N_3_ O_7_ S_2_ Na ) in an aqueous acetic acid solution (10%).

#### Proteins densitometry

To estimate more precisely the densitometry of the protein shift, the KODAK Molecular Imaging Software, V.4.5. was used, applying to digital photography of the electrophoretic profiles with the equipment (Logic 100 Imaging System), capturing the digital data of the bands, obtaining the average and the standard deviation of each lane.

## Results and discussion

### Biological material

Ten bulk samples (1 kg each) of seeds were collected and transported to the DryLab-AORTech laboratories. Seeds were manually cleaned from foreign matter, eliminating broken or immature seeds, and the moisture was measured. The results ranged from 45.05 g/dm^3^ for control samples with 14.9% *d.b.* moisture and a mass of 100 *seeds* of 7.78 ± 0.13 g, to 36.43 g/dm^3^ for samples dehydrated for 1.5 h in DEM-LP, with a moisture content of 3.92 g/dm^3^ and 100-piece mass of 6.92 g^4^.

### Dehydration methods

#### Thermo-solar system (TSD)

The internal conditions in the dehydration chamber were: the temperature oscillates between (44.1–66.4) °C and 1.00 ± 0.21 m/s air flux. After 45 min (0.75 h), the first sample was withdrawn to be analyzed, this had a moisture of 12.18% *d.b.* The second sample remained for 4 h having 2.30% moisture *d.b.* With this method, 2 h is required to reach the goal of 8% moisture (*d.b.*)^4^.

#### Dehydration by electromagnetic induction and low-pressures (DEMI-LP)

The heating chamber reaches 65 °C in 5 min, and the internal relative pressure can be reduced from the atmospheric pressure to approximately − 20 in Hg in 3 min, maintaining the chamber negative pressure concerning the atmosphere. The combination of low pressure and high temperature produces the evaporation of water, and in this process, the samples eliminate the moisture.

Samples of 1 kg at 14.95% 14.95% moisture (*d.b.*) of sunflower seeds, the first sample were dehydrated by 30 min (0.5 h) reducing to 10.43%, the second sample remained for 1 h, reaching 6.29% and the third sample was dehydrated by 1.5 h and the result was 3.92% moisture (*d.b.*), the necessary time to reach 8% of Moisture was 47 min^4^, then the all others chemical characterizations and few biological were carried out.

### High-Resolution transmission electron microscope

**Figure 1:**
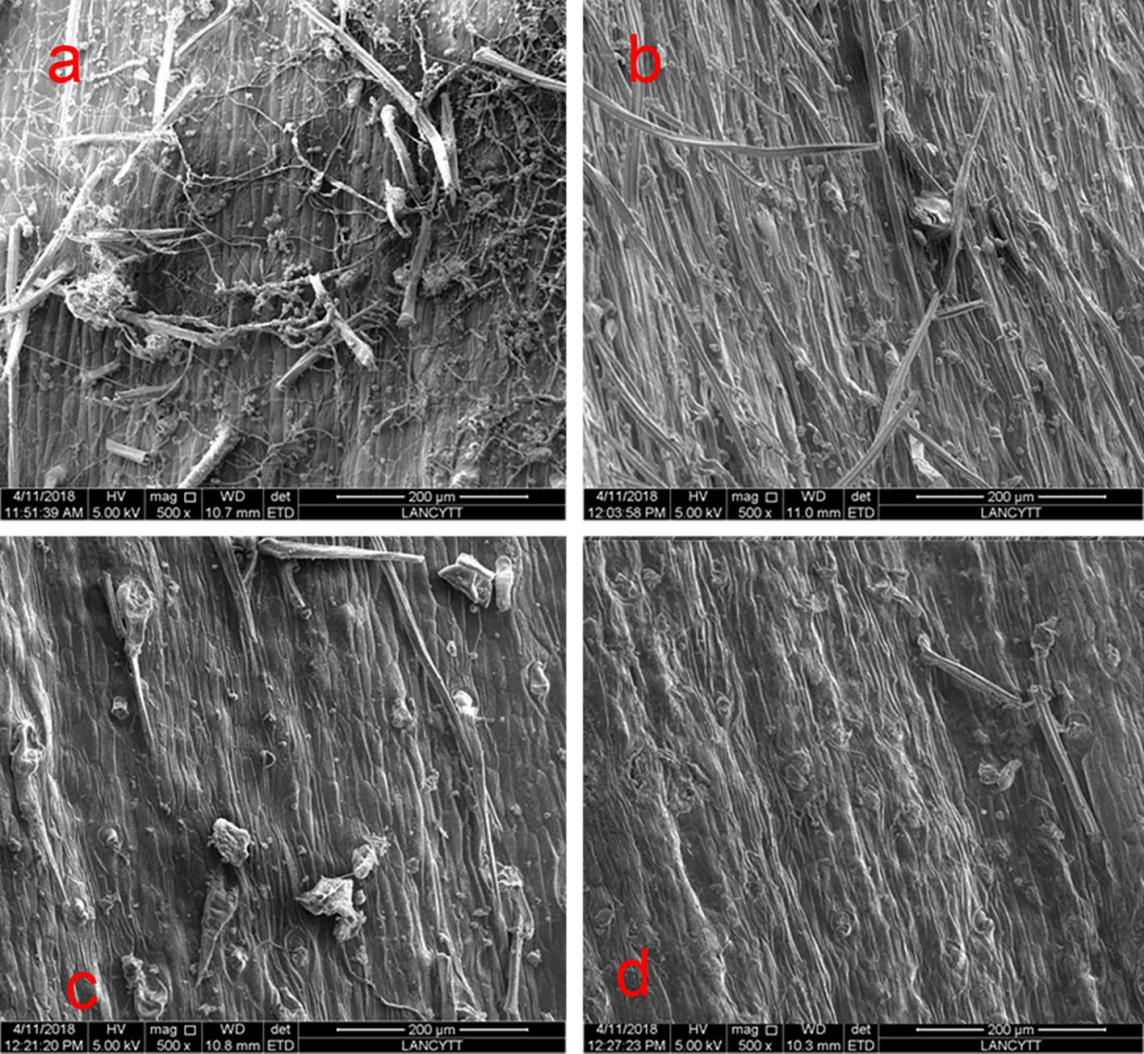
Transmission electron micrographs of sunflower seeds: (**a**) Control, (**b**) 4 h TSD, (**c**) 0.5, h and (**d**) 1 h DEMI-LP.

Figure 1, shows the micrographs of the whole seeds, there can be seen how the hull fiber thickness was evaluated by a 200 μm microphotography analysis (500×), there had some changes in thickness, these detected changes explain why the volumetric expansion of whole seeds had increased by around 5%, as reported by Ortiz-Hernandez et al.^4^.

### Viability of germination and growth

Table 1 shows the numerical results of the laboratory germination experiment, comparing the two selected drying methods and the different times, showing some interesting data, it can be seen the variation as the time of dehydration increases and moisture decreases, the germination variations are related to humidity and the relationship what time is just a consequence. This Table shows the loss of physiological quality of germination and grow viability, and also indicates that the loss of vigor occurs much faster when the germination decreases^18^.

**Table 1:** Averages of the viability percentage for sunflower seeds according to the dehydration treatment used: Thermo-solar (TSD), Electromagnetic Induction, and Low Pressure (DEMI-LP).

Sample	Total seeds	Not viable (%)	Viable (%)
Control	25	34	66
0.5 h DEMI-LP	25	34	66
1.0 h DEMI-LP	25	60	40
4 h TSD	25	76	24

The viability of seeds dehydrated for 0.5 h with the DEMI-LP method was the same as the control 66%, reducing to 40% after 1 h of dehydration. The lowest viability was obtained in seeds dehydrated for 4 h using the TSD method, just 24%.

### Bromatological analysis

The percentual results obtained in the bromatological determination of the sunflower seeds are shown in Table 2. Even though the seeds were dehydrated, the humidity percentage was 10.4% in the sample 0.5 h DEMI-LP, 6.29% for 1 h DEMI-LP, and 2.31% in the sample 4 h TSD.

**Table 2:** Nutritional content of sunflower seeds dehydrated by Electromagnetic induction (DEMI-LP) and Thermo-solar (TSD).

Sample	% DM	% M	% CF	% A	% P	% TRS	% EE	% TOC	Cal
Control	85.05	14.95	28.55	1.45	14.51	7.95	35.70	99.19	339.72
0.5 h DEMI-LP	89.57	10.43	29.87	3.78	6.46	8.83	31.72	97.90	283.20
1.0 h DEMI-LP	93.71	6.29	24.95	4.09	5.19	3.19	32.22	97.73	242.27
4 h TSD	97.69	2.31	29.08	1.15	5.09	3.44	33.53	99.36	255.79

Dry Matter (DM), Moisture (M), Crude Fiber (CF), Ash (A), Protein (P), Total Reducing Sugars (TRS), Ether Extract (EE), Total Organic Carbon (TOC), and Calories (Cal).

The percentage of ashes was higher in the sample 1 h DEMI-LP (4%), followed by 0.5 h in DEMI-LP (3.7%), and the lowest concentration was obtained in 4 h TSD (1.1%). This parameter indicates the amount of inorganic matter present in the samples^14^.

In Table 2 In accord with the results of nutrimental content in the sunflower seeds *EE* content was 35.7% in the control, decreasing in the dehydration treatments being the most notorious in the sample 0.5 h DEMI-LP with 31.7%.

The *TOC* content presented a maximum average value of 99.3% in 4 h by *TSD* and a minimum average of 97.7% in 1 h, in *DEMI-LP*. The importance of *TOC* derives from the decomposition of plants because it is used as a purity test on which bacterial growth and metabolic activities of living organisms or chemical compounds will depend as mentioned by Hendricks^19^, that is, the metabolic activity of the sunflower seed was not altered by the treatments used in dehydration.

### Mineral analysis (macro and micro)

Table 3 presents the numerical results about minerals content, where the minerals concentrations in seeds are important for animal nutrition due to their vital function in the body. In the sunflower seed, the mineral contribution stands out due to its high content of *P*, *K*, *Fe,* and *Zn*.

**Table 3:** Minerals present in sunflower seeds, according to the dehydration treatment used, Thermo-solar (TSD) and Electromagnetic Induction (DEMI-LP).

Sample	Ca (%)	P (%)	Mg (%)	K (%)	Cu (ppm)	Zn (ppm)	Mn (ppm)	Fe (ppm)
Control	0.25	1.10	0.45	1.24	23.00	130.50	32.50	95.00
0.5 h DEMI-LP	0.30	0.82	0.39	1.12	22.00	90.50	28.00	89.50
1.0 h DEMI-LP	0.26	1.07	0.45	1.26	31.50	122.00	32.50	111.50
4 h TSD	0.32	0.87	0.39	1.20	22.50	107.50	32.50	159.50

(Ca) Calcium, (P) Phosphorous, (Mg) Magnesium, (K) Potassium, (Cu) Cooper, (Zn) Zinc, (Mn) Manganese, (Fe) Iron.

Regardless of the dehydration method, *Ca* concentration in the control was 0.25% and increased as the dehydration time rose, on the other hand, the concentration of *P* decreased in the process of dehydration concerning the control (1.1%). This is probably due to the *P* volatility features. Phosphor in sunflower seeds is beneficial for the regulation of nerve impulses and intervenes in muscle activity, recommending its use to athletes or people who perform physical activities constantly^20^.

Iron is a mineral that occurs in high concentrations in dehydration treatments. In this analysis, the highest amount of *Fe* occurred in 4 h TSD (159.50 ppm). In comparison with *Fe*, the *Zn* concentration was lower after dehydration concerning the control (130.50 ppm). The nutritional requirement in ruminants can be covered through the consumption of sunflower seeds^21,22^.

### Extraction and quantification of proteins.

The extraction of the polypeptide part of the proteins present in the hull and the kernel was determined; their concentrations are shown in Table 4.

**Table 4:** Concentration of proteins obtained from sunflower seeds.

Sample	Concentration hull (μg/μg)	Concentration kernel (μg/μg)
Control	0.190	0.434
0.5 h DEMI-LP	0.127	0.430
1.0 h DEMI-LP	0.045	0.427
4 h TSD	0.020	0.412

In Table 4 the results of protein concentration are tabulated in function of type and time of dehydration, from this analysis where determined that the protein present in the hull is much lower and there is a loss drastically in both dehydration systems, 66.8% in 0.5 h and 23.7% in 1 h, boot in DEMI-LP and for TSD just keep 10.5% of the proteins in the hull. On the other hand, the concentration of proteins in the kernel control samples contain the highest concentration, with just 4 thousandths part (0.004) lower, in other words, keeps 99.1% of proteins with 0.5 h of DEMI-LP treatment while the samples with 1 h in keeps 98.4%, reduced the protein content 0.007 and for the sample with 4 h in TSD loss 0.022 µg for each microliter (μg/μg), compared with control data reaching 94.9% of the original protein content.

#### Protein electrophoresis of sunflower seeds tissues in polyacrylamide gels (PAGE-SDS)

**Figure 2:**
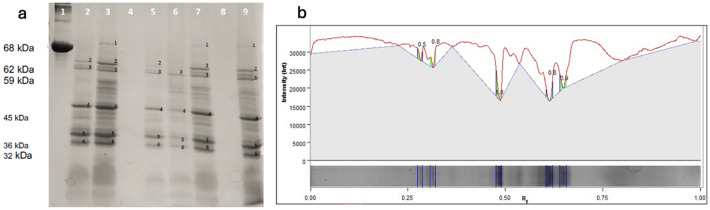
(**a**) Protein gel of sunflower seeds, control, dehydrated (0.5 and 1 h) DEMI-LP and 4 h TSD. 1) Serine bovine albumin, 68 kDa molecular weight. 2) Hull Control, 3) Kernel control, 4) Hull 4 h TSD, 5) Kernel 4 h TS, 6) Hull DEMI-LP 0.5 h, 7) Kernel DEMI-LP 1.0 h, 8) Hull DEMI-LP 1.0 h, 9) Kernel DEMI-LP 0.5 h. (**b**) Graph illustrating the percentage and absolute value of each protein fraction, based on densitometric data from lane 2 of Fig. 2a.

The electrophoretic profiles are observed in Fig. 2.

#### Proteins densitometry

In lane 1 is the 68 kDa protein used as a reference. In lanes 4, and 8, there was no expression of protein bands indicating degradation by a dehydration protein weight between 32 and 68 kDa. The protein bands can be attributed to the α-β of 11S globulin (Helianthin) as mentioned by Molina^23^.

With the results of polyacrylamide gels in the sunflower hull it is noticed that lanes 2, 4, 6, and 8 have less intensity.

In Fig. 2, the percentage of equivalence of the bands after the analysis of densitometry expressed in protein at a concentration of 60 μg/μL of each sample, the values obtained from the densitometric analysis of the peel and almond bands of the sunflower seed are observed; the analysis of the data indicated a better comparison and a numerical appreciation of the bands. The total percentage of each lane in the bands is 100%. Observing from the almond sample in lane 3, the band of protein 4 has the highest percentage (24%) followed by bands 5 and 6 (20.7% and 16.1% respectively).

The protein densitometry numerical values are shown in Table 5.

**Table 5:** Percentage of equivalence of the bands after the analysis of densitometry expressed in protein at a concentration of 60 μg / μL of each sample.

Protein bands (kDa)	Lane	1	3	5	7	9
		Albumin	Kernel Control	Kernel TSD 4 h	Kernel DEMI-LP 1 h	Kernel DEMI-LP 0.5 h
68	Percentage (%)	100.0 ± 0	6.9 ± 3.6	0.0 ± 0.0	0.9 ± 0.3	1.2 ± 0.5
62		0.0 ± 0	16.9 ± 6.8	0.0 ± 0.0	19.0 ± 3.4	17.7 ± 5.4
59		0.0 ± 0	14.4 ± 0.9	38.0 ± 11.4	23.0 ± 1.2	23.1 ± 10.8
45		0.0 ± 0	24.0 ± 3.4	26.6 ± 3.2	18.6 ± 4.1	25.1 ± 11.2
36		0.0 ± 0	18.8 ± 2.2	12.8 ± 11.4	16.1 ± 2.5	13.1 ± 3.9
32		0.0 ± 0	19.1 ± 7.3	22.5 ± 3.2	22.4 ± 6.6	19.9 ± 7.4

During dehydration, proteins were under high temperatures and pressure being the probable cause of protein denaturation, where the data indicates that the amount of oxygen present in the process is very important.

## Conclusions

This obtained data can be useful because the nutrimental content is a very important aspect to consider in food quality, and here are our interpretations of the results, in the electrophoresis study, it was found that the time on the DEMI-LP keeps protein concentration in the kernel, 99.1% in 0.5 h and 98.4% for 1 h; while in the TSD, more proteins were degraded and lost reaching just 94.9%. Then the sunflower kernel could therefore be used in the elaboration of products with greater added value, destined for human feeding, while the protein present in the hull is much lower (less than 10%) and there are lost drastically in both dehydration systems, 66.8% in 0.5 h and 23.7% in 1 h boot in DEMI-LP and for TSD just keep 10.5%.

The low degradation of proteins in DEMI-LP can be explained in accord with Morscher et al., the amount of oxygen present in the dry process has an important role in protein oxidation^24^, another relevant point in the quality of seeds is the viability of germination and grow, in this case, the dehydration treatments affected this parameter, the less affectation by the dehydration method was DEMI-LP due in 0.5 h of the treatment 66% of seeds keep the viability of the control samples, the poor germination in the analyzed samples can be attributed to the typical dormant sunflower similar to other commercial seeds^25^.

Then according to the obtained results, Dehydration type and treatment time affect germination percentages, in the equipment DEMI-LP 0.5 did not affect, maintaining 66%, and before 1 h, the percentage reduced to 40% of germinated, while in the TSD system, the germination decrease to 24% after 4 h of treatments, this data ca explain that no just the temperature is important to preserve the germination power, in accord with Morscher et al., the amount of oxygen present in the dry process, takes an important role in viability due the proteins oxidation^24^.

The chemical composition that was found showed that the minerals increased until the water concentration fell and the crude proteins decreased. The carbohydrate study showed that the best results are in 0.5 h increasing from 7.95 to 8.83%. After this, the carbohydrate concentration fell by 3.19% in 1 h.

In future research linked to the present study, considerating international quality standards established by reputable organizations in the industry. These standards, such as those set by the German Society for Fat Science (DGF) in 1988, the American Society of Petrochemists (AOCS) in 1993, the International Organization for Standardization (ISO) in 1988, and the Federation of Oil Associations, seeds and fats (FOSFA) in 1998, provide a solid foundation for assessing and comparing the quality of oilseed products internationally. Exploring how their work aligns with these standards and how they can improve to meet them in future research would be a valuable approach to further advancing this field.

## Data Availability

All data generated or analysed during this study are included in this published article [and its supplementary information files].
